# Deciphering the circRNA-Mediated ceRNA regulatory network in dendritic cells during H37Ra and BCG infection

**DOI:** 10.3389/fmolb.2026.1764518

**Published:** 2026-04-13

**Authors:** Xiaohong Sun, Shuangshuang Bao, Kaixin Zhou, Yaqi Sun, Qian Gao, Yan Lin

**Affiliations:** College of Basic Medical Sciences, Beihua University, Jilin, China

**Keywords:** ceRNA network, dendritic cells, expression profile data, high throughput analysis, *Mycobacterium tuberculosis*

## Abstract

**Background:**

Tuberculosis (TB) is a chronic infectious disease caused by *Mycobacterium tuberculosis* (*M.tb*), and poses a significant threat to global human health. Dendritic cells (DCs) represent the most potent antigen-presenting cells and play a critical role in host defenses against *M.tb* infection.

**Methods:**

We analyzed GEO datasets to identify differentially expressed circRNAs and mRNAs in *M.tb*-infected DCs. The downstream miRNAs were predicted using the circBank and CircInteractome databases, while the target mRNAs were predicted using TargetScan and miRanda. A ceRNA regulatory network consisting of 10 circRNAs, 41 miRNAs, and 145 mRNAs was constructed. Protein–protein interaction (PPI) analysis identified 30 hub nodes, which were further subjected to Gene Ontology and KEGG enrichment analyses.Subsequently, the circRNA/miRNA/mRNA regulatory axis was assessed, followed by validation using qRT-PCR and evaluation through ROC curve analysis.

**Results:**

Four core genes—*STAT1*, *BCL2*, *TRAF6*, and *IL1A*—were enriched in tuberculosis,JAK–STAT,and NF-κB pathways. Two ceRNA axes, circNFATC3/miR-150-5p/*STAT1* and circNFATC3/miR-23a-3p/*BCL2*, were validated by qRT-PCR, showing expression patterns consistent with high-throughput data. ROC analysis demonstrated strong diagnostic potential (AUC >0.7) for both axes.

**Conclusion:**

This study constructed a ceRNA regulatory network in *M.tb*-infected DCs, identified key molecular modules, and proposed circRNA-associated axes as promising biomarkers for early TB diagnosis and potential therapeutic targets.However, these findings are limited to experimental models of DCs infection and require further validation in clinical samples and *in vivo* models.

## 1 Introduction

Tuberculosis (TB) is a chronic infectious disease caused by *Mycobacterium tuberculosis* (*M.tb*). It poses a serious threat to human health and exerts a significant impact on global public health, resulting in approximately 1.3 million deaths annually. Among infectious diseases, TB exhibits the highest mortality rate attributable to a single pathogenic agent. According to the report published by the World Health Organization, TB accounted for approximately 1.25 million deaths worldwide in 2023 (World Health Organization WHO, 2024). Following entry into the human body, *M.tb* colonizes the host through the respiratory tract and can persist within the granuloma microenvironment for several decades (Krueger et al., 2025). This period is characterized by an asymptomatic, subclinical phase during which no notable clinical manifestations are observed. However, the infection may reactivate due to aging or the progression of underlying diseases in the host, resulting in active disease characterized by clinical symptoms and increased infectivity. These characteristics complicate the timely detection and therapeutic management of TB (Ehrt et al., 2018). Recent years have witnessed substantial progress in the prevention and control of TB, driven by advancements in medical technology and strengthened global health cooperation. However, numerous challenges remain, including delays in detection and treatment, an increasing incidence of drug-resistant TB cases, and the slow development of novel therapeutic agents and technologies. Therefore, the diagnosis and prevention of TB continue to demand sustained efforts and innovative research initiatives (Boshoff et al., 2023).

Antigen-presenting cells play a crucial role in the immune responses against *M.tb* during the initial stages of infection by initiating protective immune responses (Cronan, 2022). Among these, dendritic cells (DCs) are professional antigen-presenting cells that play a critical role in the immune response against *M.tb* infection, and function as a bridge between the primary and adaptive immune responses (Kurowska-Stolarska et al., 2017). Host immune defense plays a key role in controlling *M.tb* infection. The processes of antigen presentation, cytokine secretion, apoptosis, and DCs maturation are fundamental to the resistance against *M.tb* infection (Rodrigues et al., 2020; Espinosa-Cueto et al., 2017; Liu et al., 2020).

Recent studies have demonstrated that non-coding RNA (ncRNAs) play critical roles in regulating the host immune response, and the circRNA-miRNA-mRNA regulatory network has been extensively characterized in a variety of tumors. However, research on host immune responses to *M.tb* infection remains relatively limited (Chen et al., 2022; Zhang M. et al., 2021; Liang et al., 2020). CircRNAs, a class of ncRNAs, directly influence mRNA transcription and regulate the expression of target genes by acting as miRNA sponges, thereby playing a central role in the immune response against *M.tb* infection (Kazemi et al., 2023; Wang et al., 2022; Jabeen et al., 2024). Research has demonstrated that circTRAPPC6B, which is downregulated in the peripheral blood mononuclear cells (PBMCs) of patients with active TB, antagonizes the miR-874-3p-mediated suppression of *ATG16L1* expression, induces autophagy in *M.tb*-infected macrophages, and inhibits the growth of *M.tb* (Luo et al., 2021). A previous study demonstrated that circTRAPPC6B achieved an area under the curve (AUC) of 0.8609 for the diagnosis of active TB, indicating its potential utility as a novel biomarker for TB infection (Zhuang et al., 2017). The differential expression of circRNAs, miRNAs, and mRNAs plays a critical role in modulating host responses to *M.tb* infection and may serve as a promising biomarker for the diagnosis and targeted treatment of TB.

Therefore, elucidating the dynamic competing endogenous RNAs (ceRNA) regulatory network in DCs during *M.tb* infection is of critical importance. By establishing a ceRNA regulatory network and performing enrichment analyses of the core genes, this study systematically identified the key functional modules involved in DCs-mediated immune responses to *M.tb* infection. The findings provide novel theoretical insights into the pathogenesis of TB and highlight potential therapeutic targets for the effective treatment and control of disease progression.

## 2 Materials and methods

### 2.1 Acquisition and analysis of microarray datasets

Gene expression microarray datasets were retrieved from the Gene Expression Omnibus (GEO) database of the NCBI (http://www.ncbi.nlm.nih.gov/) using the search terms “tuberculosis” and “dendritic cells”, with “*Homo sapiens*” specified as the target organism. The search yielded microarray expression profiles associated with *M.tb* infection in DCs that met the predefined experimental criteria. Following screening, circRNA and mRNA expression datasets from patients with *M.tb* infection and normal healthy control subjects were selected for analysis. The original microarray (chip) dataset was retrieved, and the probe identifiers were converted to the corresponding gene symbols using the bioDBnet (https://biodbnet-abcc.ncifcrf.gov) online platform to facilitate subsequent hierarchical clustering analysis. The circRNAs and mRNAs were subjected to differential expression analysis using the GEO2R (https://www.ncbi.nlm.nih.gov/gds/?term=GEO2R) online web server, and the results were stored in Excel format for further analyses. The differentially expressed circRNAs and mRNAs were identified using the criteria: p < 0.05 and |log_2_FC| > 1, and the results were visualized in a volcano plot.

### 2.2 Construction of circRNA-miRNA-mRNA network

CircBase (https://www.circbase.org), established by Glažar et al. (2014). In 2014, is a comprehensive circRNA database dedicated to circRNAs. It provides extensive resources for circRNA research, including circRNAs identified across a wide range of organs and cell lines in various species, including humans, mice, nematodes, and fruit flies. The CircInteractome (https://circinteractome.gov) database enables users to query circRNA names, genomic coordinates, and mature circRNA sequences; predict interactions between circRNA and RNA-binding proteins (RBPs) or miRNAs; and design divergent polymerase chain reaction (PCR) primers and circRNA-specific siRNAs. TargetScan (https://www.targetscan.org) is an online database that is widely used for miRNA target prediction, and provides detailed information regarding miRNA-mediated gene regulation by analyzing the binding sites between miRNAs and their potential target genes. The 3ʹ-UTR screening approach primarily relies on sequence complementarity between miRNAs and target mRNAs, the thermodynamic stability of miRNA-mRNA duplexes, and evolutionary conservation of target sites across species. In this study, the DEcircRNA-DEmiRNA interactions were predicted using the circBase and CircInteractome databases, while the downstream target genes of key differentially expressed miRNAs were identified using the TargetScan and miRanda databases (https://www.microrna.org/microrna/). The resulting circRNA-miRNA and miRNA-mRNA interaction pairs were imported into Cytoscape (version 3.10.2) for the construction and visualization of the circRNA-miRNA-mRNA regulatory network.

### 2.3 Protein-protein interaction (PPI) network analysis and screening of core genes

The STRING database (https://cn.string-db.org/) (version 12.0) was used to construct a PPI network for the differentially expressed genes. The proteins encoded by these genes exhibit potential functional associations, and STRING assesses the strength of these associations and displays the PPIs through a network, thereby enabling the identification of core proteins within the network. Each node in the network represents a protein, while the edges connecting the nodes are depicted as lines of varying thickness and color, indicating the strength of the interactions. The complete “string_interactions.tsv” file was retrieved from the STRING database and imported into Cytoscape (version 3.10.2) to construct the initial PPI regulatory network. The core genes were selected using the cytoNCA plugin of Cytoscape based on the degree centrality (DC), betweenness centrality (BC), and closeness centrality (CC). Nodes with DCs, BC, and CC values greater than the median were retained, and the final core gene regulatory network was subsequently constructed.

### 2.4 Gene Ontology (GO) and kyoto encyclopedia of genes and genomes (KEGG) enrichment analyses

GO enrichment analysis is extensively employed to assess the functional enrichment of gene lists within specific GO categories. In this classification system, gene functions are categorized into three domains: biological process (BP), cellular component (CC), and molecular function (MF). KEGG enrichment analysis is a bioinformatics approach that utilizes the KEGG database to determine whether metabolic and signaling pathways are significantly activated or inhibited under specific experimental conditions, thereby facilitating the understanding of the biological mechanisms underlying gene expression changes. In this study, GO enrichment analysis was conducted to assess the functional enrichment of core genes in the PPI network across the CC, MF, and BP categories. KEGG pathway enrichment analysis was additionally performed to identify the significantly enriched signaling pathways associated with the core genes.

### 2.5 Cultivation of *M.tb* and DCs

A ready-to-use *M.tb* culture medium was used to cultivate the H37Ra(American Type Culture Collection, ATCC) and BCG (BeNa Culture Collection, BNCC) strains of *M.tb*. The cultures were incubated at 37 °C with continuous shaking at 150 rpm for 3–4 weeks. The optical density (OD) was measured once the culture medium became visibly turbid, and acid-fast staining was performed using the Birkmann acid-fast staining kit to confirm the presence of *M.tb*. The PBMCs(Beijing Nuopu Biological Co.,Ltd.Beijing, China) were resuspended in RPMI-1640 serum-free liquid medium by vigorous pipetting. The cell concentration was adjusted to 1 × 10^6^ cells/mL and transferred to T25 cm^2^ culture flasks. The cells were incubated at 37 °C in a 5% CO_2_ incubator for 2 h, following which the cell suspension and non-adherent cells were removed. The adherent cells were cultured in RPMI-1640 medium supplemented with 100 ng/mL granulocyte-macrophage colony-stimulating factor (GM-CSF) (Suzhou Novoprotein Technology Co., Ltd., Suzhou, China,catalog number:C003), 100 ng/mL interleukin (IL-4) (Suzhou Novoprotein Technology Co., Ltd.,Suzhou, China,catalog number:CX03), and 10% fetal bovine serum (FBS) (GIBCO, United States). Half of the volume of the culture medium was replaced on days 2 and 4, and 20 ng/mL tumor necrosis factor (TNF-α)(Suzhou Novoprotein Technology Co., Ltd., Suzhou, China, catalog number:C008)was added from day 5 onward to promote the maturation of DCs (Meskini et al., 2024; Ackaert et al., 2025; Lühr et al., 2020). It is worth noting that the TNF-α stimulation employed here serves as a classical inflammatory stimulus to induce DCs into a validated activated state under *in vitro* conditions. This does not fully replicate the physiological differentiation or maturation processes of dendritic cells (DCs) *in vivo*.

### 2.6 Phenotypic identification of dendritic cells

Cells were harvested on day 1 and day 8 of culture, respectively. After washing twice with phosphate-buffered saline (PBS), the cells were centrifuged at 300 *g* for 5 min, and the supernatant was discarded. The cell pellet was resuspended in cell staining buffer (Elabscience Biotechnology Co.,Ltd.,Wuhan, China) and adjusted to a concentration of 4 × 10^6^ cells/mL. A 100 µL aliquot of the cell suspension was transferred into flow cytometry tubes as an unstained control. Subsequently, 100 µL of the cell suspension was separately incubated with 5 µL of APC Anti-Human CD1a Antibody and PE Anti-Human CD83 Antibody 5 µL of antibodies (Elabscience Biotechnology Co., Ltd., Wuhan, China).The samples were mixed thoroughly and incubated for 30 min at room temperature in the dark. After incubation, the cells were washed once with cell staining buffer and resuspended in 400 µL of the same buffer. Fluorescence signals were then analyzed using a flow cytometer (Beckman Coulter, Inc., United States) to assess dendritic cell surface marker expression.

The following lasers and filters were used: At 638 nm laser red 660/20 was used for APC detection. At 488 nm laser the yellow 585/42 filter was used for PE detection.

### 2.7 Laser confocal microscopy

The H37Ra and BCG strains of *M.tb* were initially washed thrice with phosphate-buffered saline (PBS) buffer and subsequently resuspended in carbonate buffer. Fluorescein isothiocyanate (FITC, from Good Laboratory Practice Bioscience, GLPBIO) was then added to the bacterial suspension at a final concentration of 50 μg/mL, followed by incubation in the dark for 2 h. Following incubation, the mixture was centrifuged at 5,000 rpm for 10 min and the supernatant was discarded. The precipitate was then washed thrice with PBS and resuspended in RPMI-1640 medium at a final concentration of 1 × 10^6^ cells/mL. The cells were inoculated onto cell slides placed in six-well plates for a 24-h infection period. The lysosomal components of the DCs were labeled with LysoTracker Red DND-99 (GLPBIO) at a concentration of 70 nM and incubated at 37 °C in the dark for 90 min, following which the working solution was removed. The cells were subsequently fixed with 4% paraformaldehyde for 20 min, washed with PBS for 2 min, and finally mounted with an anti-fade mounting medium.

### 2.8 Enzyme-linked immunosorbent assay (ELISA)

The expression levels of TNF-α and IL-1β(both from Wuhan GeneBeauty Biotechnology Co., Ltd., Wuhan, China) in DCs infected with the H37Ra and BCG strains of *M.tb* were determined by ELISA. Briefly, supernatants from the *M.tb*-infected and control groups were collected at 6, 12, and 24 h post-infection, and centrifuged at 3,000 rpm for 20 min. Samples were collected in triplicate at each time point. The procedures were conducted in accordance with the instructions provided with the ELISA kit, following which the absorbance was measured at 450 nm using an enzyme-labeled microplate reader (Molecular Devices-SpectraMax ABS, United States). Baseline calibration was performed using the blank control prior to the sequential measurement of OD values from designated wells.

### 2.9 Detection of mRNA, miRNA, and circRNA expression levels by quantitative reverse transcription (qRT)-PCR

Total RNA was extracted using the FastPure Cell Total RNA Isolation Kit, while miRNA was isolated using the miRcute miRNA Isolation Kit (Vazyme, Nanjing, China).The purity and concentration of the RNA samples were assessed using a microplate spectrophotometer (Thermo Fisher Scientific, Massachusetts, United States), following which the samples were stored at −80 °C for further analyses. The expression levels of mRNAs and circRNAs were quantified using the ChamQ Universal SYBR qPCR Master Mix (Vazyme, Nanjing, China) on a ProFlex PCR system (Thermo Fisher Scientific, Massachusetts, United States). *β-actin* was used as the internal reference gene for quantifying the relative expression levels of the differentially expressed mRNAs and circRNAs using the 2^−ΔΔCt^ method. The expression levels of miRNAs were quantified using the miRcute Plus miRNA qPCR Kit (SYBR Green)(Tiangen, Beijing, China) on a ProFlex PCR(Thermo Fisher Scientific, Massachusetts, United States) system. *U6* was used as the internal reference gene for quantifying the relative expression levels of the differentially expressed miRNAs using the 2^−ΔΔCt^ method. The qRT-PCR data were analyzed using GraphPad Prism (version 10.0). A t-test was performed to assess the between-group differences, and p < 0.05 was considered statistically significant.

### 2.10 Receiver operating characteristic (ROC) curve analysis

The differences in the expression levels of *STAT1*, *BCL2*, miR-150-5p, miR-23a-3p, and circNFATC3 between the control and *M.tb*-infected groups were determined by ROC curve analysis. ROC curves were generated using GraphPad Prism (version 10.0), and the sensitivity, specificity, and AUC values were calculated. Binary logistic regression analysis was conducted using SPSS (version 19.0), followed by ROC curve analysis to evaluate whether the ceRNA axis as a whole was associated with the clinicopathological processes of *M.tb*-infected DCs. The sensitivity, specificity, and AUC values were determined, and p < 0.05 was considered to be statistically significant.

### 2.11 Statistical analysis

All the data of this experiment were statistically analyzed using ImageJ and GraphPad Prism (version 8.0) software. When comparing between two samples, the T-test was used, while for multiple sample comparisons, the One Way ANOVA was employed. The statistical analysis results were all represented by the mean ± standard deviation (Mean ± SD). In the statistical graphs, * indicated a statistically significant difference (P < 0.05), ** indicated a significant difference (P < 0.01), and *** indicated a highly significant difference (P < 0.001). Moreover, all the experimental data were verified by at least three experiments.

## 3 Results

### 3.1 Identification of differentially expressed circRNAs and mRNAs

**FIGURE 1:**
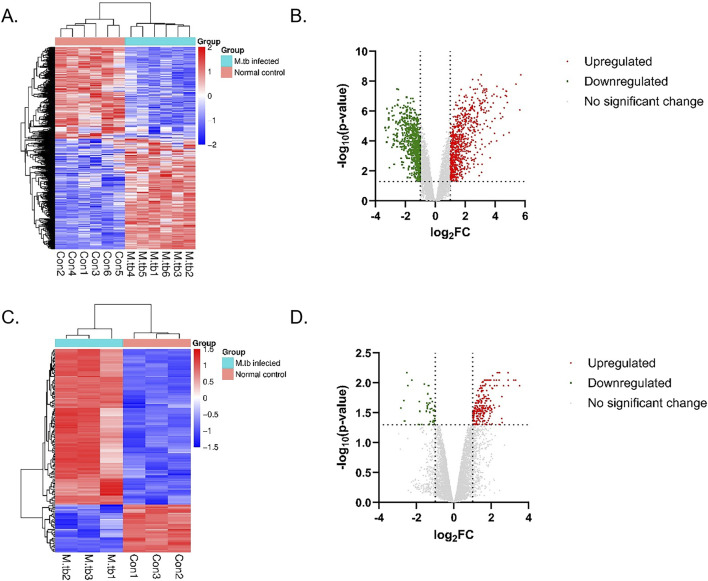
Differential expression analysis and visualization of mRNAs and circRNAs. **(A)** Hierarchical clustering analysis of the differentially expressed mRNAs in the GSE34151 dataset. **(B)** Volcano plot depicting the differentially expressed mRNAs in the GSE34151 dataset. **(C)** Hierarchical clustering analysis of the circRNAs in the GSE117563 dataset. **(D)** Volcano plot depicting the differentially expressed circRNAs in the GSE117563 dataset.The red and green circles denote upregulated and downregulated expression, respectively, and the gray circles represent no statistically significant difference in expression.

The mRNA expression dataset (GSE34151) and circRNA expression dataset (GSE117563) that met the predefined screening criteria were retrieved from the GEO database and served as the original data sources. A total of 1388 differentially expressed mRNAs were identified from the GSE34151 mRNA expression dataset based on the screening criteria: |log_2_FC| > 1 and p < 0.05, of which 736 were upregulated and 652 were downregulated (Supplementary Table S1). A total of 283 differentially expressed circRNAs were identified from the GSE117563 circRNA expression dataset, including 244 upregulated and 39 downregulated circRNAs (Supplementary Table S2). The GSE34151 mRNA dataset and the GSE117563 circRNA dataset were analyzed through hierarchical clustering. In the resulting heatmaps, each row corresponds to an mRNA or circRNA, while each column represents a sample from the *M.tb*-infected group or the uninfected control group. The red regions indicate higher expression levels in the *M.tb*-infected group relative to those in the control group, whereas the blue regions denote higher expression levels in the control group compared to those in the *M.tb*-infected group (Figures 1A,C). Hierarchical clustering analysis clearly delineated the differential expression patterns of the circRNAs and mRNAs. The 283 differentially expressed circRNAs and 1388 mRNAs, identified based on the screening criteria |log_2_FC| > 1 and p < 0.05, were represented using a volcano plot (Figures 1B,D).

### 3.2 Construction of circRNA-miRNA-mRNA regulatory network

**FIGURE 2:**
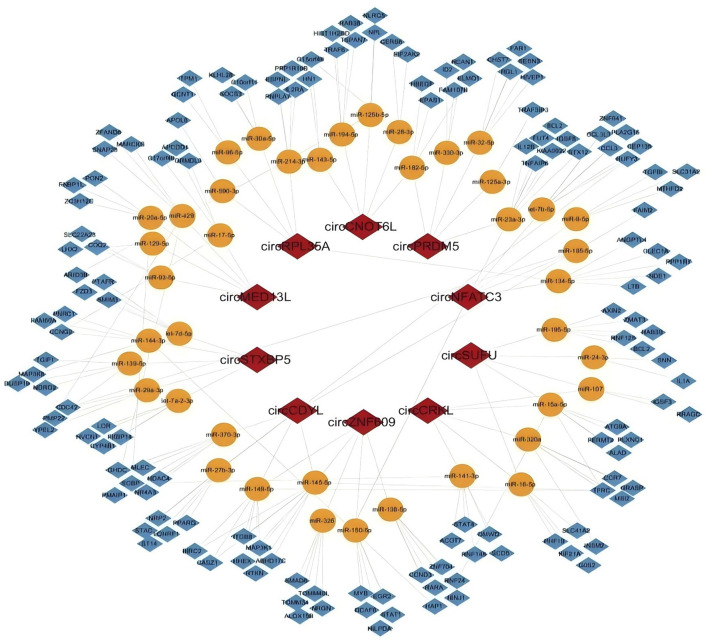
Construction of the circRNA–miRNA–mRNA regulatory network.The red diamonds represent circRNAs, the orange circles represent miRNAs, the blue diamonds denote mRNAs, and the gray solid lines connecting the nodes depict the regulatory relationships among circRNAs, miRNAs, and mRNAs.This network illustrates the putative ceRNA regulatory interactions and served as the basis for the identification of key regulatory axes associated with dendritic cell responses to *Mycobacterium tuberculosis* infection.

To explore the potential regulatory roles of circRNAs in *M.tb*-infected DCs, the five most significantly upregulated and five most significantly downregulated circRNAs were selected based on the magnitude of their fold changes. Utilising the circBank and circInteractome databases to predict their downstream miRNAs, employing the Targetscan and miRanda databases to predict differentially expressed mRNAs. The predicted target genes were cross-referenced with mRNA expression data from *M.tb*-infected DCs for constructing the circRNA-miRNA-mRNA ceRNA regulatory network (Figure 2). The resulting regulatory network consisted of 10 circRNAs, 41 miRNAs, and 145 mRNAs (Supplementary Table S3), indicating that circRNAs may regulate gene expression by acting as miRNA sponges and thereby modulating downstream mRNA targets.

### 3.3 PPI network analysis and screening of core genes

**FIGURE 3:**
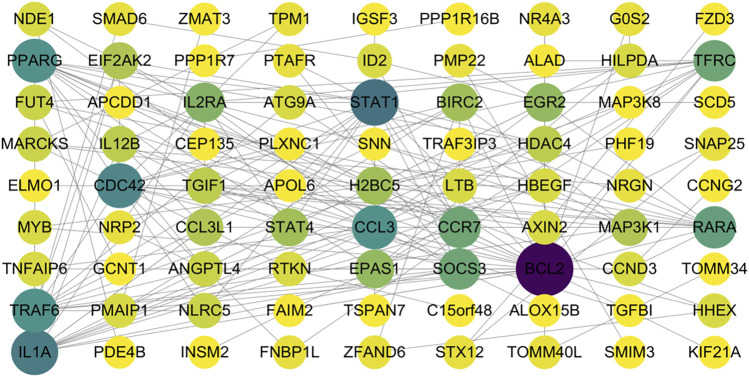
Construction of the PPI network for differentially expressed genes.Each node represents a protein and each edge represents a protein–protein interaction.The node size and color intensity indicate the connectivity degree of each protein within the network, with darker colors representing higher degrees of interaction. This network illustrates the potential interactions among differentially expressed genes and provides insight into their functional associations.

**FIGURE 4:**
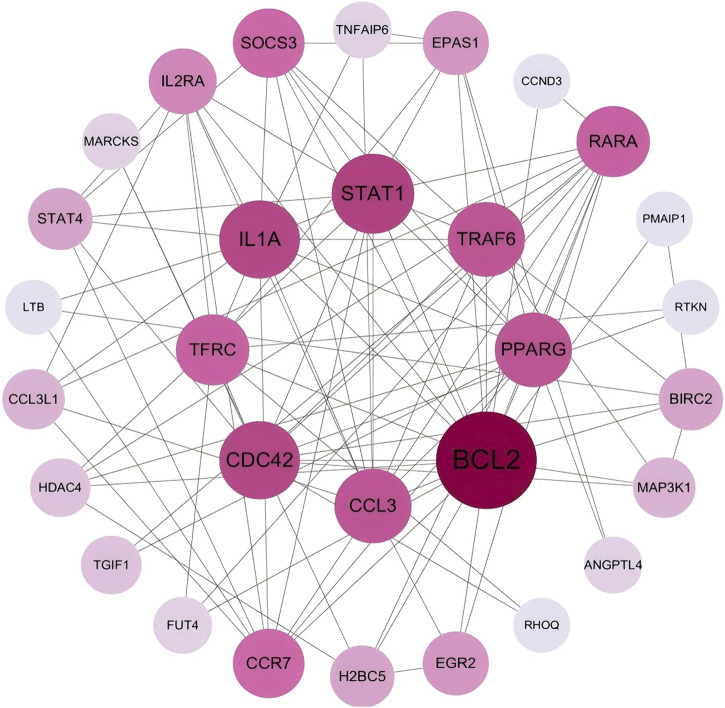
PPI network of the 30 core genes selected from the differentially expressed genes.In this network, larger nodes represent genes with higher connectivity and potential biological importance. Hub genes such as *BCL2*, *STAT1, TRAF6* and *IL1A* exhibited high connectivity, suggesting their potential roles in the regulatory network.

The mRNAs within the regulatory network were subjected to PPI network analysis to construct a PPI network of differentially expressed genes. The PPI network of differentially expressed genes was constructed using the STRING database, where each node represents a protein, and the thickness and color intensity of the edges correspond to the strength of the interactions. The interactions between the nodes were visualized using Cytoscape software, and the initial PPI network diagram was generated (Figure 3). To further delineate the key regulatory elements, 30 core genes within the PPI regulatory network, including *BCL2*,*STAT1*,*TRAF6,IL1A*, and others, were identified based on three centrality measures (DC, BC, and CC) using the cytoNCA plugin of Cytoscape (Figure 4). The results of DC, BC and CC of differentially expressed genes are shown in Table 1.

**TABLE 1:** Analysis results of DC, BC, and CC.

Name	Betweenness	Closeness	Degree
*BCL2*	2039.731496	0.072943172	27
*STAT1*	410.3004592	0.071487947	17
*IL1A*	350.4090472	0.071369295	16
*CDC42*	898.1346113	0.071310116	16
*PPARG*	799.8875672	0.071133168	14
*TRAF6*	403.1285094	0.070781893	14
*CCL3*	292.3350129	0.07101569	14
*TFRC*	658.3916357	0.070723684	12
*RARA*	621.4975543	0.071487947	12
*CCR7*	283.3985738	0.070549631	11
*SOCS3*	100.5052752	0.070549631	11
*IL2RA*	62.01785423	0.070204082	9
*EPAS1*	340.3077183	0.070607553	8
*EGR2*	496.8976912	0.070146819	8
*H2BC5*	256.1980519	0.069918699	7
*BIRC2*	187.4261905	0.07008965	7
*STAT4*	37.68399542	0.069076305	7
*MAP3K1*	290	0.069918699	6
*CCL3L1*	9.997691198	0.068855084	6
*TGIF1*	576.7888889	0.068745004	5
*HDAC4*	14.54545455	0.069918699	5
*ANGPTL4*	300.3649598	0.068037975	4
*FUT4*	154.65	0.069635628	4
*TNFAIP6*	148.8770288	0.068091845	4
*MARCKS*	177.2309651	0.068091845	4
*PMAIP1*	146	0.068965517	3
*CCND3*	146	0.06918745	3
*LTB*	7.685714286	0.068199841	3
*RTKN*	146	0.068199841	3
*RHOQ*	8.044397759	0.0681458	3

### 3.4 GO and KEGG enrichment analyses

**FIGURE 5:**
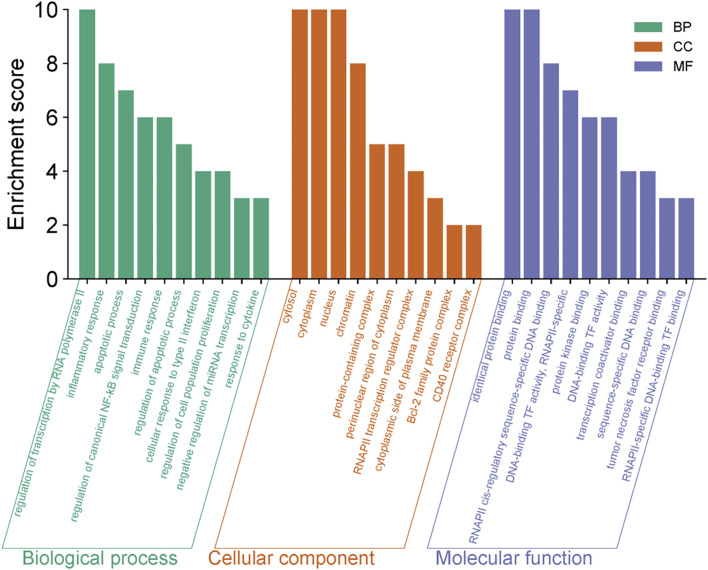
Functional annotation analysis of the core target genes, presented as a bar graph depicting the significantly enriched GO terms across the BP,CC and MF categories.The horizontal axis represents functional entries across the BP, CC, and MF categories, while the vertical axis denotes enrichment scores. Longer bars indicate a greater number of genes enriched for that functional entry, signifying a more significant corrected p-value.

**FIGURE 6:**
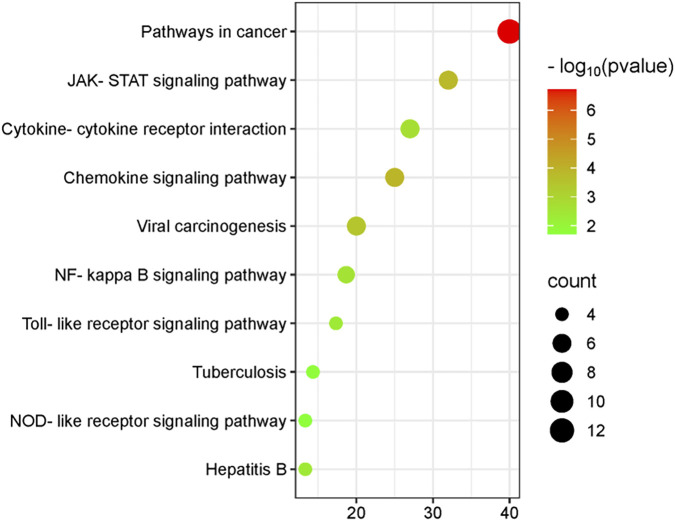
Bubble plot depicting the significantly enriched KEGG pathways associated with the target genes.The horizontal axis represents the enrichment factor, while the size of the circle denotes the count. A larger circle indicates a greater number of genes enriched within this signalling pathway, whereas a redder circle signifies a more significant corrected p-value.

The 30 core genes identified from the PPI network were subjected to comprehensive functional and pathway enrichment analyses using the DAVID database (https://davidbioinformatics.nih.gov/), including GO functional annotation and KEGG pathway analyses, to elucidate their biological functions and involvement in key signaling pathways. The findings revealed that the core genes were significantly enriched in several GO terms in the BP category, including positive regulation of transcription by RNA polymerase II, inflammatory response, apoptotic process, and immune response. The significantly enriched GO terms in the CC category primarily included the cytosol, cytoplasm, and nucleus, while the significantly enriched GO terms in the MF category predominantly included protein binding and specific binding to the cis-regulatory region of RNA polymerase (Figure 5). KEGG pathway enrichment analysis revealed that four core genes, namely, *STAT1*, *BCL2*, tumor necrosis factor receptor-associated factor (*TRAF*), and *IL1A*, were significantly enriched in both the tuberculosis pathway and the Janus kinase (JAK)-STAT signaling pathway and NF-kappa B signaling pathway. These findings suggest that these genes play critical roles in the immune responses of DCs against *M.tb* infection, thereby providing a robust foundation for further research (Figure 6). Detailed enrichment information for GO and KEGG pathways is presented in Tables 2,3.

**TABLE 2:** Important GO classification terms of 30 core genes.

GO ID	Term	P-value	Associated genes found
GO:0045944	Regulation of transcrip-tion by RNA polymerase II	3.81E-07	*DAC4, IL1A, EGR2, RARA, STAT1, TRAF6, STAT4* *EPAS1, PPARG, RHOQ*
GO:0006954	Inflammatory response	5.56E-06	*HDAC4, IL1A, TNFAIP6* *CCL3L1, IL2RA, CCL3* *CCR7, FUT4*
GO:0006915	Apoptotic process	4.54E-04	*IL1A, MARCKS, RTKN* *IL2RA, BCL2, PMAIP1* *BIRC2*
GO:0043123	Regulation of canonical NF-kappaB signal	4.53E-04	*IL1A, TFRC, TRAF6* *CCR7, LTB, BIRC2*
GO:0006955	Immune response	3.50E-04	*IL1A, CCL3L1, IL2RA* *CCL3, CCR7, LTB*
GO:0005829	Cytosol	8.40E-04	*STAT1, CDC42, IL1A, SOCS3, CCND3, TRAF6, CCL3, BCL2, STAT4, RARA*
GO:0005737	Cytoplasm	8.32E-04	*EGR2, MAP3K1, EPAS1* *STAT1, CDC42, TRAF6, CCL3, BCL2, STAT4* *RARA*
GO:000078	Chromatin	7.15E-04	*TGIF1, EGR2, EPAS1* *STAT1, IL1A, CCND3, TRAF6, BCL2, STAT4, RARA*
GO:0032991	Rotein-containing complex	4.23E-04	*TGIF1, HDAC4, EGR2* *EPAS1, STAT1, STAT4* *RARA, PPARG*
GO:0042802	Identical protein binding	5.04E-05	*CDC42, TFRC, STAT1* *TRAF6, BCL2, STAT4* *CCL3, PPARG, ANGPTL4* *BIRC2*
GO:0005515	Protein binding	2.27E-05	*TFRC, EPAS1,CDC42, CCL3, MAP3K1, STAT1, IL1A* *TRAF6, BCL2, RARA*
GO:0000978	RNA polymerase II ci-regulatory sequence-specific DNA binding	9.98E-04	*TGIF1, HDAC4, EGR2* *EPAS1, STAT1, STAT4* *RARA, PPARG*
GO:0000981	DNA-binding TF activi-ty, RNA II-specific	8.08E-04	*TGIF1, EGR2, EPAS1, STAT1, STAT4, RARA, PPARG*
GO:0019901	Protein kinase binding	1.26E-04	*CDC42, HDAC4, CCND3, MAP3K1, TFRC, RHOQ*

**TABLE 3:** Important KEGG classification terms of 30 core genes.

GO ID	Term	P-value	Associated genes found
hsa05200	Pathways in cancer	1.86E-07	*CDC42, CCND3, EPAS1* *STAT1, TRAF6, IL2RA* *BCL2, STAT4, RARA* *PMAIP1, PPARG, BIRC2*
hsa04630	JAK-STAT signaling pathway	3.44E-04	*SOCS3, CCND3, STAT1* *IL1A, BCL2, STAT4*
hsa04060	Cytokine-cytokine receptor interaction	3.40E-04	*IL1A, CCL3L1, IL2RA* *CCL3, CCR7, LTB*
hsa04062	Chemokine signaling pathway	1.29E-04	*CDC42, IL1A, STAT1* *CCL3, CCR7,BCL2*
hsa05203	Viral carcinogenesis	1.17E-04	*CDC42, HDAC4, CCND3* *EGR2, H2BC5, PMAIP1*
hsa04064	NF-kappa B signaling pathway	0.002607665	*TRAF6, BCL2, IL1A, STAT1,MAP3K1*
hsa04620	Toll-like receptor signaling pathway	0.003371495	*CCL3L1, STAT1, TRAF6* *CCL3*
hsa05152	Tuberculosis	0.003800605	*IL1A, STAT1, TRAF6, BCL2*
hsa05161	Hepatitis B	0.006960702	*STAT1, TRAF6, BCL2, STAT4*
hsa04621	NOD-like receptor signaling pathway	0.009296126	*STAT1, TRAF6, BCL2, BIRC2*

### 3.5 Expression of CD1a and CD83 on dendritic cells

**FIGURE 7:**
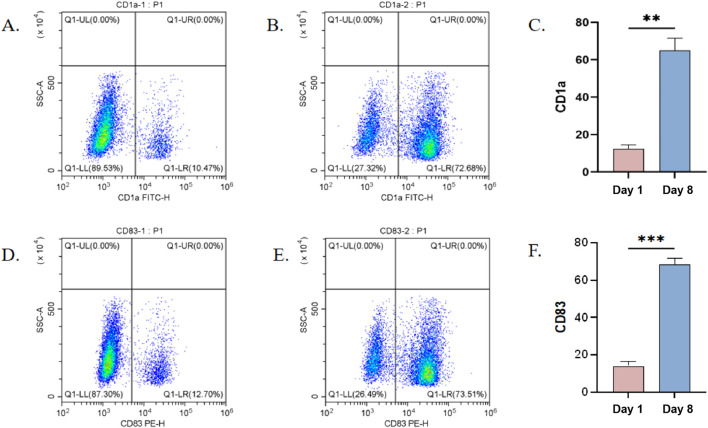
Flow cytometric analysis of CD1a and CD83 expression on the surface of DCs at different time points. **(A)** Expression of CDla on Day 1 of Culture; **(B)** Expression of CDla on Day 8 of Culture; **(C)** Statistical Analysis Results of CDla on Day 1 and Day 8 of Culture; **(D)** Expression of CD83 on Day 1 of Culture; **(E)** Expression of CD83 on Day 8 of Culture; **(F)** Statistical Analysis Results of CD83 on Day 1 and Day 8 of Culture. It is evident that the maturation rate of DCs induced by GM-CSF and IL-4 has been significantly enhanced, thereby facilitating sample preparation for subsequent experiments.Statistical significance is indicated in the figure (*p < 0.05, **P < 0.01, ***P < 0.001).

To verify the maturation status of DCs.Flow cytometry was used to analyze the expression levels of the cells surface markers CD1a and CD83 on DCs. DCs were collected on day 1 and day 8 of culture and incubated with fluorescently labeled anti-CD1a and anti-CD83 antibodies. The results showed that, compared to day 1 DCs, the expression of CD1a and CD83 was significantly increased in DCs cultured for 8 days, exhibiting a typical dendritic cell immune phenotype (Figure 7).

### 3.6 Laser confocal microscopy of *M.tb*-infected DCs

**FIGURE 8:**
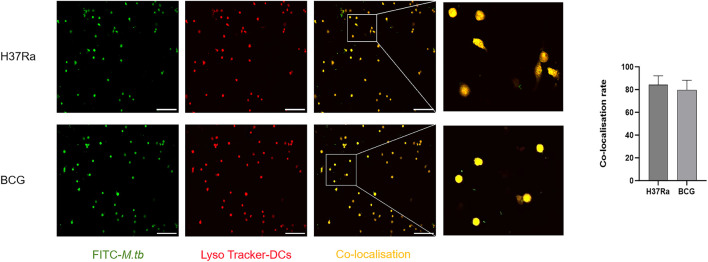
Representative confocal laser microscopy images (scale:50 μm) and co-localization rates of DCs infected with the H37Ra and BCG strains of *M.tb*.Representative confocal laser scanning microscopy images showing FITC-labeled mycobacteria (green), LysoTracker-labeled lysosomes in DCs (red), and their merged co-localization signals (yellow) in DCs infected with the H37Ra or BCG strains. Enlarged boxed regions are shown to highlight representative co-localization events. The bar graph on the right summarizes the co-localization rates in the two infected groups. Both H37Ra-infected and BCG-infected DCs exhibited substantial co-localization of mycobacteria with lysosomes.

DCs were infected with the attenuated H37Ra and BCG strains of *M.tb*, and the cells labeled with FITC-conjugated *M.tb* exhibited green fluorescence, whereas those labeled with LysoTracker Red DND-99 displayed red fluorescence. The co-localization of green and red fluorescence following simultaneous excitation produced yellow fluorescence due to the overlap of fluorescent signals, indicates that *M.tb* has successfully infected DCs and been phagocytosed by them. Based on morphological assessment, the infection model has been successfully established. Morphological validation of the infection model revealed co-localization efficiencies greater than 75% across all samples (Figure 8).

### 3.7 Expression levels of pro-inflammatory factors TNF-α and IL-1β

**FIGURE 9:**
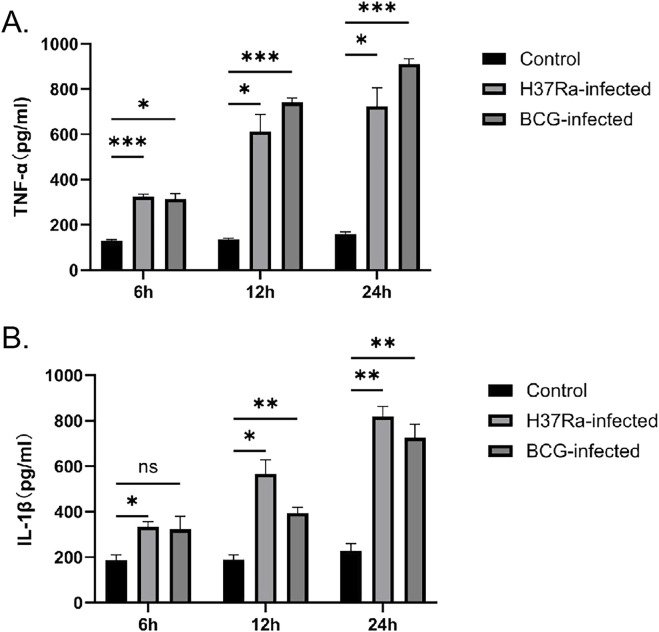
H37Ra and BCG infection promotes time-dependent secretion of pro-inflammatory cytokines by DCs. Secretion levels of **(A)** TNF-α and **(B)** IL-1β were measured in the culture supernatants of DCs from the control, H37Ra-infected, and BCG-infected groups at 6, 12, and 24 h post-infection. Both H37Ra and BCG infection increased the secretion of TNF-α and IL-1β compared with the control group, with the highest levels observed at 24 h. Statistical significance is indicated in the figure (*p < 0.05, **P < 0.01, ***P < 0.001).

To verify the successful establishment of the DCs infection model, the secretion levels of the pro-inflammatory cytokines TNF-α and IL-1β were measured at different time points after *M.tb* infection. Research findings indicate that both the H37Ra-infected group and the BCG-infected group exhibited elevated secretion of TNF-α and IL-1β compared to the control group, with this increase becoming more pronounced over time. Peak cytokine levels were observed 24 h post-infection, demonstrating that *M.tb* stimulation elicited a robust inflammatory response within DCs, thereby confirming the successful establishment of the infection model. (Figure 9). Therefore, samples were collected 24 h post-infection for subsequent experiments.

### 3.8 Quantitative validation of key mRNAs expression by qRT-PCR

**FIGURE 10:**
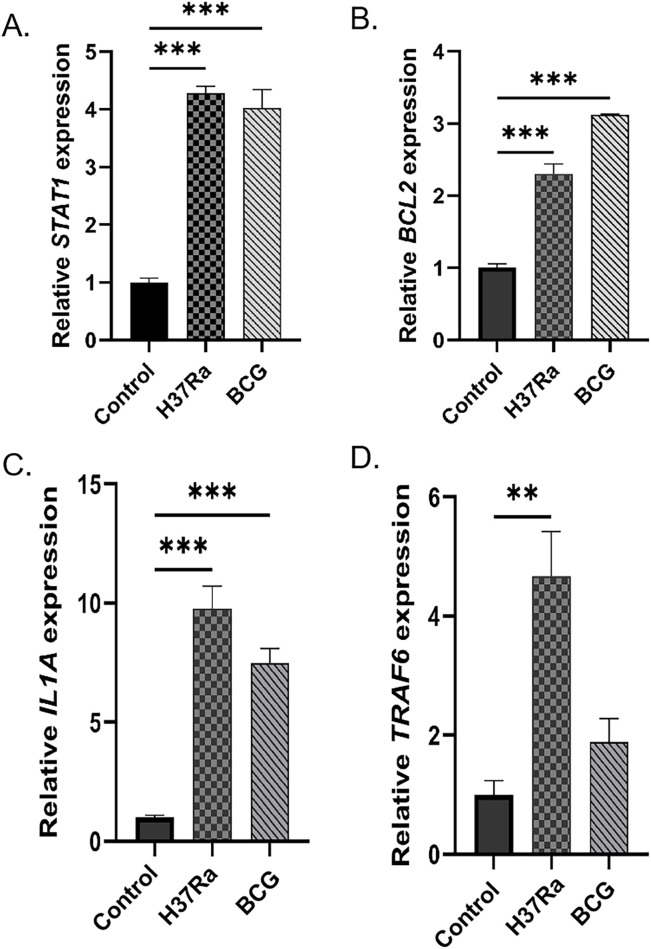
qRT-PCR validation of key mRNAs associated with the tuberculosis pathway. Relative mRNA expression levels of **(A)** STAT1, **(B)** BCL2, **(C)** IL1A, and **(D)** TRAF6 in DCs from the control, H37Ra-infected and BCG-infected groups. Statistical significance is indicated in the figure (*p < 0.05, **P < 0.01, ***P < 0.001).

Enrichment analysis of the mRNAs in the ceRNA regulatory network revealed that the mRNA expression levels of *STAT1*, *BCL2*, *IL1A*, and *TRAF6* were significantly enriched in the tuberculosis pathway. Subsequent validation by qRT-PCR revealed that the mRNA expression levels of *STAT1*, *BCL2*, and *IL1A* were significantly upregulated in the *M.tb*-infected group, consistent with the results of microarray analysis (Figures 10A–C). As illustrated in Figure 9, the expression of *TRAF* mRNA was upregulated in DCs infected with the H37Ra strain; however, there were no significant differences in *TRAF* expression between the BCG-infected and control groups (Figure 10D). Combining GO functional enrichment and KEGG pathway analysis results, *STAT1*, *BCL2*, and *IL1A* were identified as potential key regulatory genes in the process of *Mycobacterium tuberculosis* infection of DCs, providing crucial evidence for subsequent mechanistic studies.

### 3.9 Quantitative validation of key miRNAs expression by qRT-PCR

**FIGURE 11:**
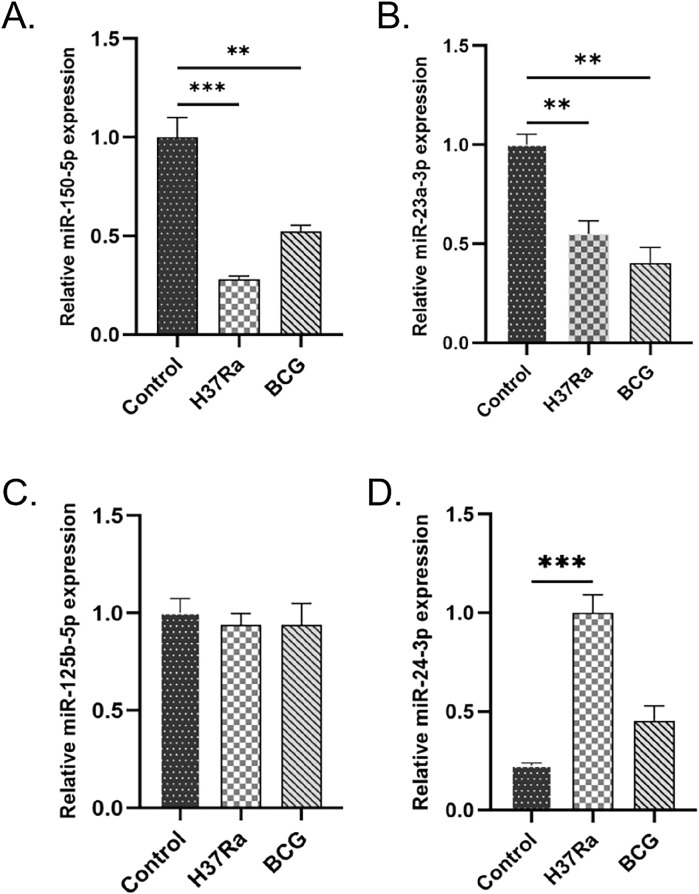
qRT-PCR validation of key miRNAs identified from the ceRNA regulatory network. Relative expression levels of **(A)** miR-150-5p, **(B)** miR-23a-3p, **(C)** miR-125b-5p, and **(D)** miR-24-3p in DCs from the control, H37Ra-infected and BCG-infected groups.Statistical significance is indicated in the figure (*p < 0.05, **P < 0.01, ***P < 0.001).

The miRNAs that exhibited inverse expression patterns within the same ceRNA axis as *STAT1*, *BCL2*, and *IL1A*—identified from the circRNA-miRNA-mRNA regulatory network—were further evaluated using qRT-PCR. A total of 4 miRNAs, namely, miR-150-5p, miR-24-3p, miR-23a-3p, and miR-125b-5p, were selected for qRT-PCR analysis. The findings revealed that miR-150-5p and miR-23a-3p were significantly downregulated in the *M.tb*-infected groups (Figures 11A,B). However, no significant difference in miR-125b-5p expression was detected between the infected and control groups (Figure 11C). Additionally, although miR-24-3p expression was upregulated in the H37Ra-infected group, there was no significant difference in expression between the BCG-infected and control groups (Figure 11D).

### 3.10 Quantitative validation of key circRNAs expression by qRT-PCR

**FIGURE 12:**
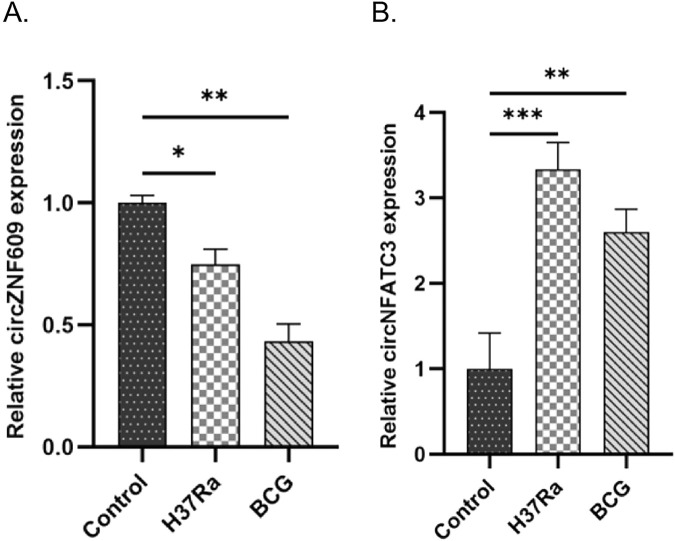
qRT-PCR validation of key circRNAs identified from the ceRNA regulatory network. Relative expression levels of **(A)** circZNF609 and **(B)** circNFATC3 in DCs from the control, H37Ra-infected and BCG-infected groups.Statistical significance is indicated in the figure (*p < 0.05, **P < 0.01, ***P < 0.001).

The circRNAs that exhibited inverse expression patterns within the same ceRNA axis as miR-150-5p and miR-23a-3p—identified from the circRNA-miRNA-mRNA regulatory network—were validated by qRT-PCR. Specifically, the expression levels of two circRNAs, namely, circZNF609 and circNFATC3, were analyzed. The findings revealed that circNFATC3 expression was upregulated in the *M.tb*-infected group, whereas circZNF609 expression was downregulated. The expression pattern of circNFATC3 was consistent with the results of microarray analysis, whereas the expression of circZNF609 deviated from the microarray findings. The relative expression levels of circZNF609 and circNFATC3 in the *M.tb*-infected and control groups are illustrated (Figure 12).

### 3.11 Evaluation of molecular diagnostic performance

**FIGURE 13:**
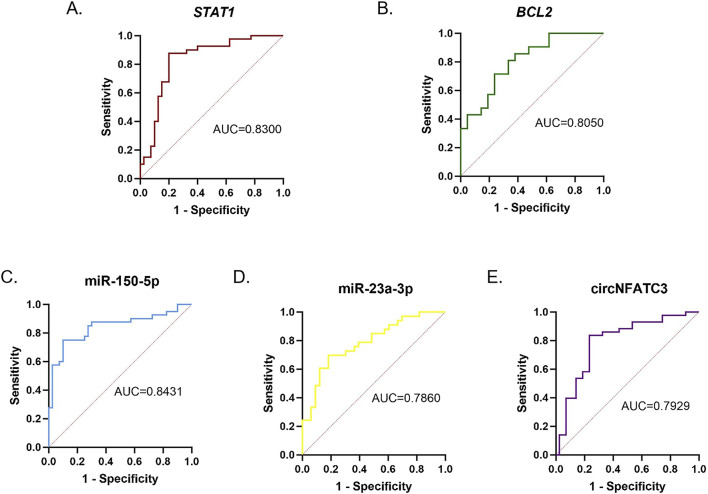
ROC curves illustrating the diagnostic performance of **(A)** *STAT1*, **(B)** *BCL2*, **(C)** miR-150-5p, **(D)** miR-23a-3p, and **(E)** circNFATC3 in the diagnosis of TB.

**FIGURE 14:**
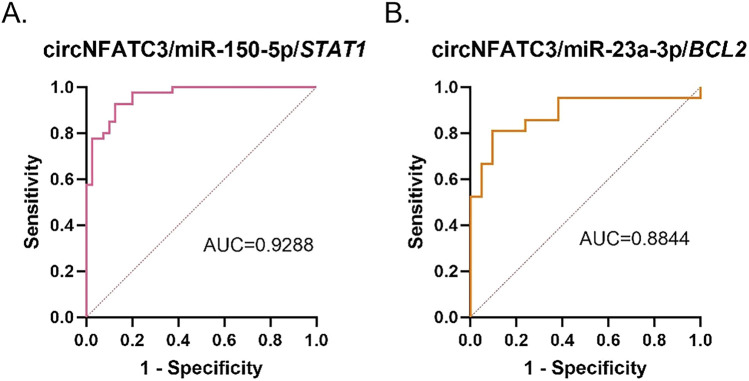
ROC curves illustrating the diagnostic performance of the **(A)** circNFATC3/miR-150-5p/*STAT1* and **(B)** circNFATC3/miR-23a-3p/*BCL2* axes in the diagnosis of TB.

The AUC value serves as a robust and comprehensive metric for evaluating the diagnostic accuracy of clinical tests. In this study, ROC curves were generated for *STAT1*, *BCL2*, miR-150-5p, miR-23a-3p, and circNFATC3 based on their relative expression levels, and their associations with the clinical characteristics of *M.tb*-infected DCs were analyzed. The findings revealed that the AUC, sensitivity, and specificity of *STAT1* were 0.8300, 78.57%, and 82.13%, respectively, while those of *BCL2* were 0.8050, 78.16%, and 77.31%, respectively (Figures 13A,B). The AUC, sensitivity, and specificity of miR-150-5p were 0.8431, 86.60%, and 76.52%, respectively, while those of miR-23a-3p were 0.7860, 76.57%, and 80.00%, respectively (Figures 13C,D). The AUC, sensitivity, and specificity of circNFATC3 were 0.7929, 84.43%, and 73.33%, respectively (Figure 13E). The circNFATC3/miR-150-5p/*STAT1* and circNFATC3/miR-23a-3p/*BCL2* ceRNA axes demonstrated AUC values of 0.9288 and 0.8844, respectively, with sensitivities of 91.86% and 82.33%, respectively, and specificities of 86.71% and 90.81%, respectively. The higher specificity and sensitivity of the ceRNA axes suggest that the expression patterns of the circNFATC3/miR-150-5p/*STAT1* and circNFATC3/miR-23a-3p/*BCL2* axes may serve as potential biomarker clusters for characterizing the progression of *M.tb* infection in DCs (As shown in Figure 14; Table 4).

**TABLE 4:** Estimation of diagnostic value of ROC curve.

Gene	AUC	Sensitivity (%)	Specificity (%)	P value
*STAT1*	0.8300	78.57	82.13	0.0012
*BCL2*	0.8050	78.16	77.31	0.0078
miR-150-5p	0.8431	85.60	76.52	0.0026
miR-23a-3p	0.7860	76.57	80.00	0.0046
circNFATC3	0.7929	84.43	73.33	0.0014
*STAT1*/miR-150-5p/circNFATC3	0.9288	91.86	82.33	0.0001
*BCL2*/miR-23a-3p/circNFATC3	0.8844	86.71	90.81	0.0004

## 4 Discussion

TB is a chronic infectious disease caused by *M.tb* and represents a significant global public health challenge. Despite ongoing advancements in diagnostic and therapeutic techniques, TB continues to exhibit high incidence and mortality rates, resulting in a substantial number of fatalities annually. The early diagnosis and treatment of TB are critical measures for controlling the sources of infection and reducing disease prevalence. However, further research is necessary for advancing the diagnosis and therapeutic strategies for TB. Recent studies demonstrate that ncRNAs play a critical role in TB infection, and their differential expression during the course of infection serves as a potential biomarker for monitoring disease progression and therapeutic response (Sinigaglia et al., 2020; Fathizadeh et al., 2020; Yang et al., 2025). However, the mechanisms underlying the role of ncRNAs in the pathogenesis of TB require further investigation.

CircRNAs have garnered significant research interest in recent years. The interaction between miRNAs and circRNAs through shared binding sites reduces the number of binding sites for mRNA binding. Additionally, circRNAs harboring multiple competitive sites compete for binding with endogenous mRNAs, thereby forming the basis of the ceRNA regulatory network (Ren et al., 2019; Pu et al., 2024). The circRNA-miRNA-mRNA regulatory network plays a significant role in the progression of TB. Although existing studies have documented the regulatory role of circRNAs in the progression of TB through the circRNA-miRNA-mRNA network, numerous circRNAs associated with the pathogenesis of TB remain to be identified. In this study, expression datasets from the GEO database were analyzed to assess circRNA and mRNA expression profiles, which led to the identification 283 circRNAs and 1,388 mRNAs that were differentially expressed in *M.tb*-infected DCs compared to that in healthy controls. The downstream genes were predicted through database analysis for constructing a comprehensive and accurate circRNA-miRNA-mRNA network.

Following network construction, enrichment analysis was performed for identifying the significantly enriched signaling pathways of the core genes, in order to predict the potential biological functions of the circRNAs within the network. The findings revealed that the genes were primarily enriched in the JAK-STAT, chemokine, and nuclear factor-kappa B (NF-κB) signaling pathways.The JAK-STAT pathway functions as a central mediator of cellular inflammation and oxidative reactions, and plays a critical role in the pathogenesis of lung diseases, including pulmonary injury and fibrosis (Montero et al., 2021).Previous studies have demonstrated that inhibiting the phosphorylation of proteins in the JAK2-STAT3 pathway reduces inflammation and alleviates pathological damage in lung tissue (Saeedi-Boroujeni et al., 2021; Yang et al., 2021). Jianfang et al. (2022). Demonstrated that the elevated expression of LINC00870 is associated with Th1/Th2-related immune responses during TB infection, mediated via the activation of the JAK/STAT signaling pathway. The anti-TB drug, delamanid, suppresses the expression of C-X-C motif chemokine ligand 10(CXCL10)by regulating the JAK2/STAT1 signaling cascade, thereby mitigating inflammation in patients with MDR-TB and offering a potential therapeutic strategy for TB cases characterized by exaggerated immune responses mediated via CXCL10 (Qiao et al., 2022). The pathology of pulmonary Tuberculosis (TB) is characterized by excessive inflammatory responses and oxidative stress. Therefore, the inhibition of the JAK-STAT pathway, regulation of inflammatory responses, and the attenuation of oxidative stress represent effective strategies for mitigating the progression of pulmonary TB. The chemokines produced during *M.tb* infection play a critical role in limiting bacterial dissemination by facilitating the migration of DCs to lymph nodes, promoting the recruitment of activated T cells to the lungs, and ensuring the proper positioning of T cells within the lung parenchyma (Monin and Khader, 2014). Although these chemokines are not capable of completely eliminating *M.tb*, they contribute to restricting its growth (Park et al., 2021). The NF-κB signaling pathway promotes the activation of pro-inflammatory factors and amplification of signal cascades to induce cellular apoptosis and inflammatory responses in several diseases (Zhou et al., 2022). Apoptosis functions as a critical host immune defense mechanism against *M.tb* infection. It has been reported that S100 calcium binding protein A4*(S100A4*) is significantly upregulated in the peripheral blood of patients with active pulmonary TB, where it promotes the BCG-induced apoptosis of Human acute monocytic leukemia cell line (THP-1)macrophages and induces the activation of the NLR family pyrin domain containing 3(NLRP3) inflammasome and NF-κB signaling pathways. The inhibition of the NF-κB or NLRP3 pathways inhibits the ability of *S100A4* to enhance the BCG-induced apoptosis of THP-1 macrophages (Li et al., 2023). CircRNAs may contribute to the maintenance of immune balance during *M.tb* infection by regulating the expression of core genes and activating the aforementioned signaling pathways, thereby influencing host cellular immunity, apoptosis, and inflammatory responses. Therefore, elucidating the specific mechanisms of the ceRNA network will provide deeper insights into the pathogenesis of *M.tb* and the regulation of host immune responses during TB infection.

In this study, four core genes enriched in the TB pathway were validated through qRT-PCR analysis. The findings revealed that the expression profiles of *STAT1*, *BCL2*, and *IL1A* were consistent with the results obtained from microarray analysis. The upstream ncRNAs that interacted with these genes were identified and validated through the established ceRNA network, revealing two ceRNA axes: circNFATC1/miR-150-5p/*STAT1* and circNFATC3/miR-23a-3p/*BCL2*. In the circNFATC3/miR-150-5p/*STAT1* axis, circNFATC3 suppresses the interaction between miR-150-5p and *STAT1* mRNA, thereby upregulating *STAT1* expression. *STAT1,* the first identified member of the STAT protein family, plays a key role in mediating immune responses and the progression of inflammatory diseases. Recent studies have demonstrated that *STAT1* plays a significant role in various pulmonary conditions, including infectious diseases, pulmonary fibrosis, pulmonary tumors, and acute lung injury (Zhang Y. et al., 2021). Current research indicates that *STAT1* and its associated signaling components may serve as potential biomarkers for *M.tb* infection (Yi et al., 2020). Negi et al. (2019) demonstrated that the activation of macrophages induces the activation of *STAT1*, which in turn increases TNF-α levels and decreases IL-10 levels, thereby inhibiting *M.tb* infection. Therefore, the role of *STAT1* in DCs-mediated immune responses to *M.tb* infection warrants further investigation and it may represent a potential therapeutic target for TB. Studies on hsa-miR-150-5p have demonstrated that it regulates the development of various types of immune cells and influences the differentiation of B cells, T cells, and natural killer cells (Ban et al., 2017). A previous study observed that miR-150-5p targets STAT1 in cardiovascular diseases to stabilize atherosclerotic plaques by modulating the proliferation and migration of human aortic smooth muscle cells in the treatment of atherosclerosis (Bian et al., 2021). Yang et al. (2023) constructed an miRNA-mRNA regulatory network and identified two key miRNAs involved in the pathogenesis of TB, namely, hsa-miR-150-5p and hsa-miR-25-3p, suggesting their potential as novel diagnostic targets for TB. To date, the regulatory functions of circNFATC3 in disease progression remain inadequately investigated. Yan et al. (2021) identified the role of the circNFATC3/miR-23b-3p/*RAI14* axis in gastric cancer cells, demonstrating that the upregulation of retinoic acid induced 14 (*RAI14*)and circNFATC3 expression, along with the downregulation of miR-23b-3p expression, correlates with poor patient prognosis. Additionally, *RAI14* has been shown to enhance the proliferation and invasion of gastric cancer cells. Analysis of the gene regulatory network constructed in this study revealed that the expression levels of *STAT1* and circNFATC3 were upregulated, whereas miR-150-5p expression was downregulated in *M.tb-*infected DCs, potentially indicating the activation of DCs during the progression of TB. However, the precise mechanism by which the circNFATC3/miR-150-5p/*STAT1* axis contributes to the pathogenesis of TB warrants further investigation.

In the circNFATC3/miR-23a-3p/*BCL2* axis, circNFATC3 inhibits the binding of miR-23a-3p to *BCL2*, thereby upregulating *BCL2* expression. BCL2 is a member of the BCL protein family and functions as an anti-apoptotic factor. The inhibition of host cell apoptosis serves as a survival strategy for *M.tb* and involves tightly regulated cellular processes and outcomes. Previous research has established that the BCL2 protein family exerts key anti-apoptotic effects by inhibiting the oligomerization of BCL-2-associated X protein/BCL2 antagonist/killer 1 (BAX/BAK) in the mitochondria-dependent apoptotic pathway, thereby representing a potential therapeutic target (Davids and Letai, 2013; Anderson et al., 2014). The targeted inhibition of the anti-apoptotic BCL2 protein leads to cellular apoptosis and represents a potential therapeutic strategy for the treatment of TB (Arnett et al., 2023). The overexpression of *BCL2* and *IL-12* in DCs increases cell survival and triggers robust antigen-specific CD8^+^ T cell responses (Zhang et al., 2020). By analyzing gene expression profiles and conducting qRT-PCR experiments, the present study confirmed that *BCL2* is upregulated in DCs following *M.tb* infection. The upregulation of *BCL2* expression may enhance the efficacy of T cell-mediated immune responses and potentially indicate a transition from innate to adaptive immunity in the host following *M.tb* infection. Previous studies have demonstrated that miR-23a-3p expression is downregulated, whereas runt related transcription factor 2 (Runx2) expression is upregulated in oral squamous cell carcinoma tissues. The overexpression of miR-23a-3p or the inhibition of Runx2 expression has been shown to suppress malignant progression in CAL-27 and TSCCA oral squamous cell carcinoma cell lines (Ma et al., 2022). Chen et al. (2020) demonstrated that miR-23a-3p expression exhibits a negative correlation with the expression of *TLR4*, *TNF-α*, *IL-6*, *IL-10*, *BCL2*, SP1 transcription factor (*SP1*), and interferon regulatory factor (*IRF1*) genes during *M.tb* infection. It has been demonstrated that miR-23a-3p expression is downregulated in patients with active pulmonary TB and high bacterial loads, and its overexpression may represent a novel host-directed immunotherapeutic strategy for active TB. CircNFATC3 has been identified as a key regulator of cell proliferation, invasion, and migration, which are closely associated with the development of cancer (Karedath et al., 2021). However, the role of the circNFATC3/miR-23a-3p/*BCL2* axis in the pathogenesis of TB infection remains poorly understood, warranting further investigation into its precise underlying mechanisms.Furthermore, for miR-24-3p, which belongs to the same miR-23a∼27a∼24-2 gene cluster, an opposite expression trend was observed under infection conditions. We propose that although belonging to the same gene cluster and typically co-transcribed to produce the same pre-miRNA, under infection conditions this may indicate post-transcriptional ‘decoupling’ regulation of cluster members within the inflammatory or infectious microenvironment. This phenomenon likely stems from differences in processing efficiency by Drosha/DGCR8 and Dicer for distinct hairpin structures within the cluster, coupled with variations in half-life and degradation rates among mature miRNAs under infectious stress. Overall, the opposing changes in miR-23a-3p and miR-24-3p more likely reflect the complexity of infection-associated post-transcriptional regulation rather than a simple imbalance in co-transcriptional consistency (Chhabra et al., 2010; Vilimova and Pfeffer, 2023; Ma et al., 2016).

The pathogenesis of TB is complex and multifactorial, involving the regulatory effects of several ncRNAs and genes. CeRNA regulatory networks provide a robust foundation for subsequent experimental studies, in-depth investigations into the pathogenesis of TB, and the identification of prognostic genes, holding significant implications for the diagnosis, treatment, and prevention of TB. Nevertheless, the present study has certain limitations, which are discussed hereafter. First, it primarily relied on big data analysis and lacks sufficient validation through clinical trials. Second, although the circRNAs, miRNAs, and mRNAs in the ceRNA network were validated through qRT-PCR, further experimental validation through dual-luciferase reporter assays, as well as overexpression and inhibition studies, is necessary for elucidating the precise mechanism by which the ceRNA axis contributes to the pathogenesis of TB.

Finally, this study also has some limitations.Although this study yielded certain mechanistic insights using a TNF-α pretreated DCs model, it is important to recognise that this model possesses limitations in terms of physiological relevance. TNF-α pretreatment can only partially mimic the activated state of DCs within an inflammatory environment and does not fully reflect the functional characteristics of immature DCs within the complex *in vivo* microenvironment. Therefore, the conclusions of this study are mainly applicable to the mechanism exploration under specific experimental conditions. Future research necessitates further validation of these findings in immature DCs models, or even in more physiologically relevant *in vivo* systems, to enhance the physiological significance and clinical translational value of the results. Furthermore, limitations exist in verifying DCs phagocytosis of *M.tb* As imaging evidence primarily relies on two-dimensional confocal observations, and bacterial suspensions may exhibit some degree of aggregation during infection, it remains challenging to entirely exclude the influence of cell surface adhesion signals on result interpretation. In future studies, we shall further optimise the dispersion and homogenisation of bacterial suspensions. This will be combined with high-magnification z-stacks or three-dimensional reconstruction and flow cytometric quantification to more rigorously distinguish surface binding from intracellular phagocytosis, thereby deepening our understanding of the underlying mechanisms.

## 5 Conclusion

This study established a ceRNA regulatory network consisting of 10 circRNAs, 41 miRNAs, and 145 mRNAs, and identified four core genes, namely, *STAT1*, *BCL2*, *TRAF6*, and *IL1A*, that were significantly enriched in the tuberculosis pathway. The expression levels of key ceRNAs within the regulatory network were validated through qRT-PCR, which revealed that the differential expression patterns of the circNFATC3/miR-150-5p/*STAT1* and circNFATC3/miR-23a-3p/*BCL2* axes were consistent with the results obtained from high throughput analysis. ROC curve analyses revealed that the circNFATC3/miR-150-5p/*STAT1* and circNFATC3/miR-23a-3p/*BCL2* axes exhibited strong correlations with the clinicopathological features of DCs-mediated immune responses to *M.tb* infection. These axes represent promising biomarker clusters for characterizing the immunological processes associated with *M.tb* infection in DCs.

## Data Availability

The original contributions presented in the study are included in the article/Supplementary Material, further inquiries can be directed to the corresponding author.

## Supplementary material

SUPPLEMENTARY TABLE S1 Information about differentially expressed mRNAs in DCs infected with M.tb.

SUPPLEMENTARY TABLE S2 Information about differentially expressed circRNAs in DCs infected with M.tb.

SUPPLEMENTARY TABLE S3 circRNA-miRNA–mRNA regulatory network information regarding the immune response of DCs to M.tb.
